# Closure of Post-thrombotic Iliac Arteriovenous Fistulas by Iliac Vein Recanalization

**DOI:** 10.1177/15266028221113745

**Published:** 2022-08-18

**Authors:** Oliver Schlager, Florian Wolf, Markus Mueller, Michael E. Gschwandtner, Christian Loewe, Renate Koppensteiner, Dietrich Beitzke, Andrea Willfort-Ehringer

**Affiliations:** 1 Division of Angiology, Department of Medicine II, Medical University of Vienna, Vienna, Austria; 2 Division of Cardiovascular and Interventional Radiology, Department of Bioimaging and Image-Guided Therapy, Medical University of Vienna, Vienna, Austria

**Keywords:** post-thrombotic syndrome, arteriovenous fistula, coiling, embolization, venous stent

## Abstract

**Purpose:**

The purpose of this study was to report the closure of iliac arteriovenous fistulas associated with a post-thrombotic iliac vein occlusion by iliac venous stent recanalization.

**Case Report:**

An 80-year-old woman presented with a worsening painful swelling of her left leg after an iliofemoral deep vein thrombosis 6 months ago. Duplex ultrasound and magnetic resonance venography revealed a post-thrombotic obstruction of her iliac veins as well as several arteriovenous fistulas between branches of her left external and internal iliac arteries and adjacent diseased venous segments. In a first attempt, coil embolization did not sustainably close these iliac arteriovenous fistulas. Direct stent recanalization of the chronically diseased iliofemoral venous segment, however, resulted in an immediate closure of arteriovenous shunt flow and subsequent improvement of clinical symptoms. Six months after iliac vein stent recanalization, still no fistulas could be detected any more, venous stents were fully patent, and the patient was free of symptoms.

**Conclusion:**

Post-thrombotic iliofemoral obstructions might be associated with the development of arteriovenous fistulas. Direct stent recanalization of the chronically occluded veins results in closure of related arteriovenous fistulas.

**Clinical Impact:**

This case suggests that the combined occurrence of post-thrombotic venous obstructions with arteriovenous fistulas, which are related to aforementioned venous lesions, should be evaluated for primary venous stent recanalization rather than fistula embolization.

## Introduction

An 80-year-old woman presented with a worsening painful swelling of her left leg. Six months before, the patient experienced an acute iliofemoral vein thrombosis, which had been treated conservatively according to the patient’s preference at that time. The patient received oral anticoagulation with apixaban 10 mg bid for 7 days followed by continuous treatment with apixaban 5 mg bid. Despite continuous oral anticoagulation and the use of compression stockings, the patient now reported an aggravation of symptoms corresponding with a Villalta scale of 18 points and the clinical CEAP class C4. No clinical signs or symptoms of heart failure were observed.

**Figure 1.**
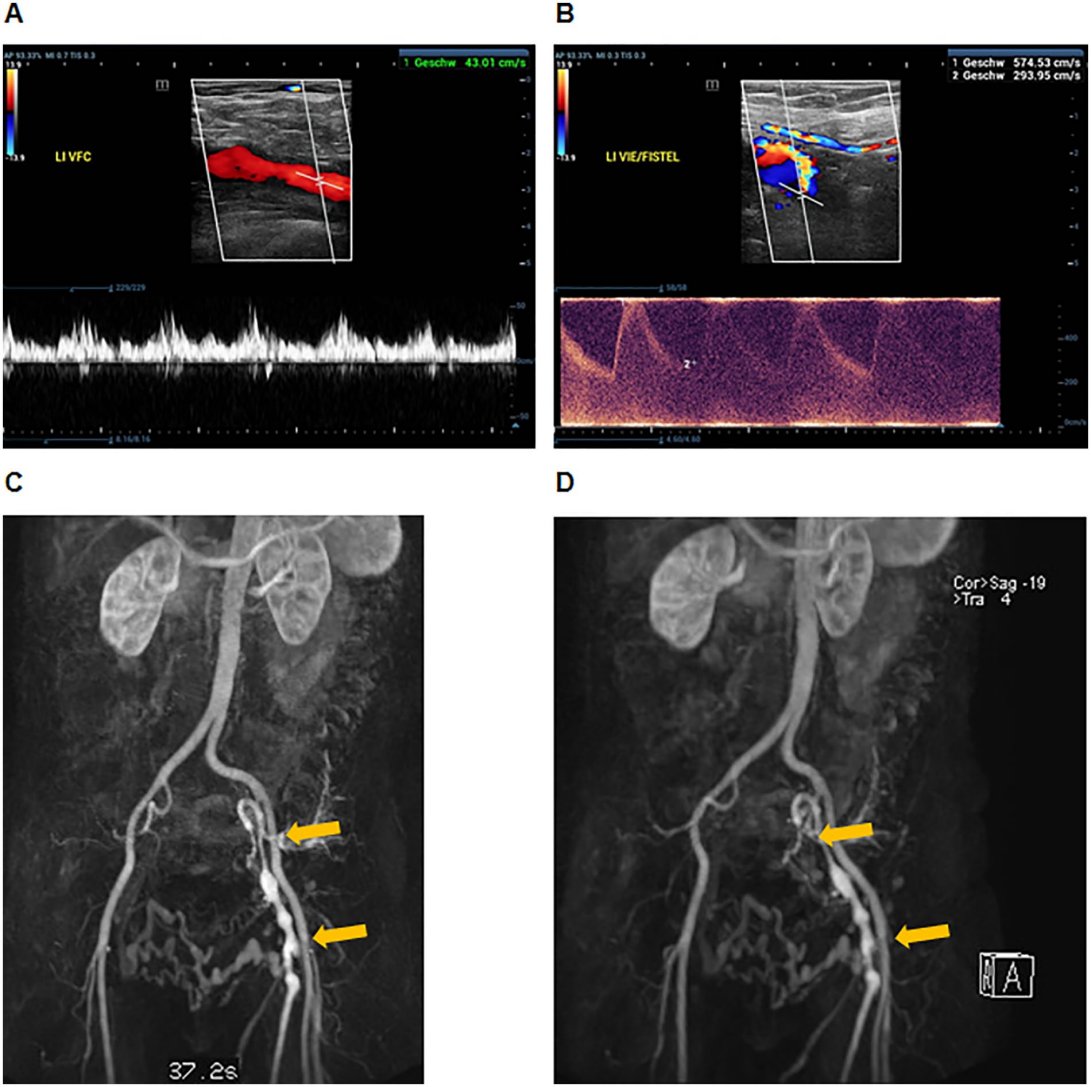
Post-thrombotic arteriovenous fistula (AVF) between the external and internal iliac artery and adjacent diseased venous segments in a patient with iliofemoral post-thrombotic syndrome. Pulsed wave doppler of the common femoral vein with arterial pulsatile flow modulation (A). AVF flow profile from a connection between the external iliac artery and vein (B). Magnetic resonance venography showing iliac AVF (orange arrows) (C and D).

Duplex ultrasound revealed post-thrombotic septae in the common femoral vein (CFV), a severe stenosis of the external iliac vein (EIV), and an occlusion of the common iliac vein (CIV). The femoral vein (FV) and the deep femoral vein (DFV) were patent. The diameter of the inferior vena cava was 20 mm without hemodynamically relevant obstructions. A point of attention was the CFV flow pattern captured by duplex, which showed an arterial pulsatile modulation caused by arteriorvenous fistulas (AVF) between the internal and external iliac arteries and adjacent diseased venous segments (Figure 1A and B). Supplementary magnetic resonance venography was used to specifically localize iliac AVF (Figure 1C and D). The etiology of these AVF was not fully clarified: there was no history of any interventional or surgical procedures involving the respective region, nor was there a history of a trauma or radiation therapy. Referring to the history of iliofemoral deep vein thrombosis in this patient, we hypothesized that these fistulas evolved as consequence of post-thrombotic scarring.

## First Approach

The pulsatile arterial flow modulation, which was captured from the CFV by Duplex ultrasound, as well as the early contrast filling of the respective CFV during the contrast enhanced, time resolved magnetic resonance venography, were conjointly indicative of a high arteriovenous shunt volume. The interdisciplinary vascular board therefore suggested an interventional 2-step approach: iliofemoral AVF had been scheduled for coil embolization in a first step followed by iliofemoral venous stent recanalization as second procedure.

## First Procedure

**Figure 2.**
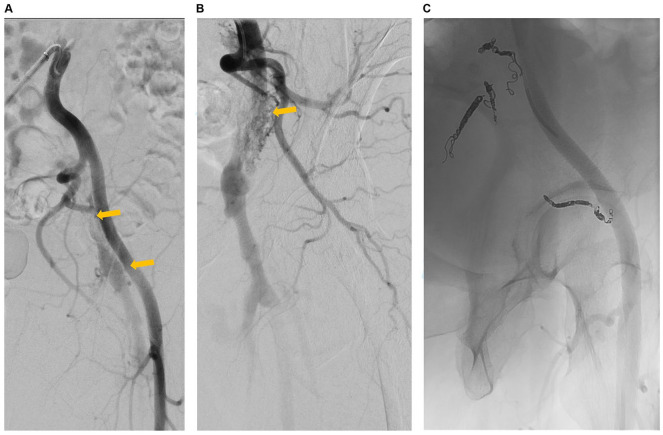
First endovascular approach with attempted coil embolization of post-thrombotic iliac arteriovenous fistula (AVF). (A) Angiography of the left iliac arteries (cross-over access from the right common femoral artery) showing venous contrast filling (orange arrows). (B) Selective angiography of left internal iliac artery showing AVF channels (orange arrow). (C) Final angiography after coil embolization.

The first procedure was the attempted embolization of AVF channels: for this approach, an arterial cross-over access via the contralateral common femoral artery was used (Figure 2A). Selective angiography of the internal iliac artery confirmed the presence of multiple AVF channels, which appeared to connect side-branches of the internal iliac artery and the post-thrombotic scarred iliac veins (Figure 2B). In total, 16 detachable coils (9 IDC soft coils, ®Boston Scientific, and 7 Concerto versa coils, ®ev3) were implanted in the respective AVF channels. Furthermore, additional AVF channels between the medial circumflex femoral artery and the CFV were identified and subsequently treated by coil embolization (2 IDC soft coils, Boston Scientific, and 3 Concerto versa coils, ev3). In the final angiogram, no further AVF channels could be identified (Figure 2C). Anticoagulation treatment was suspended after embolization.

## Clinical Course

**Figure 3.**
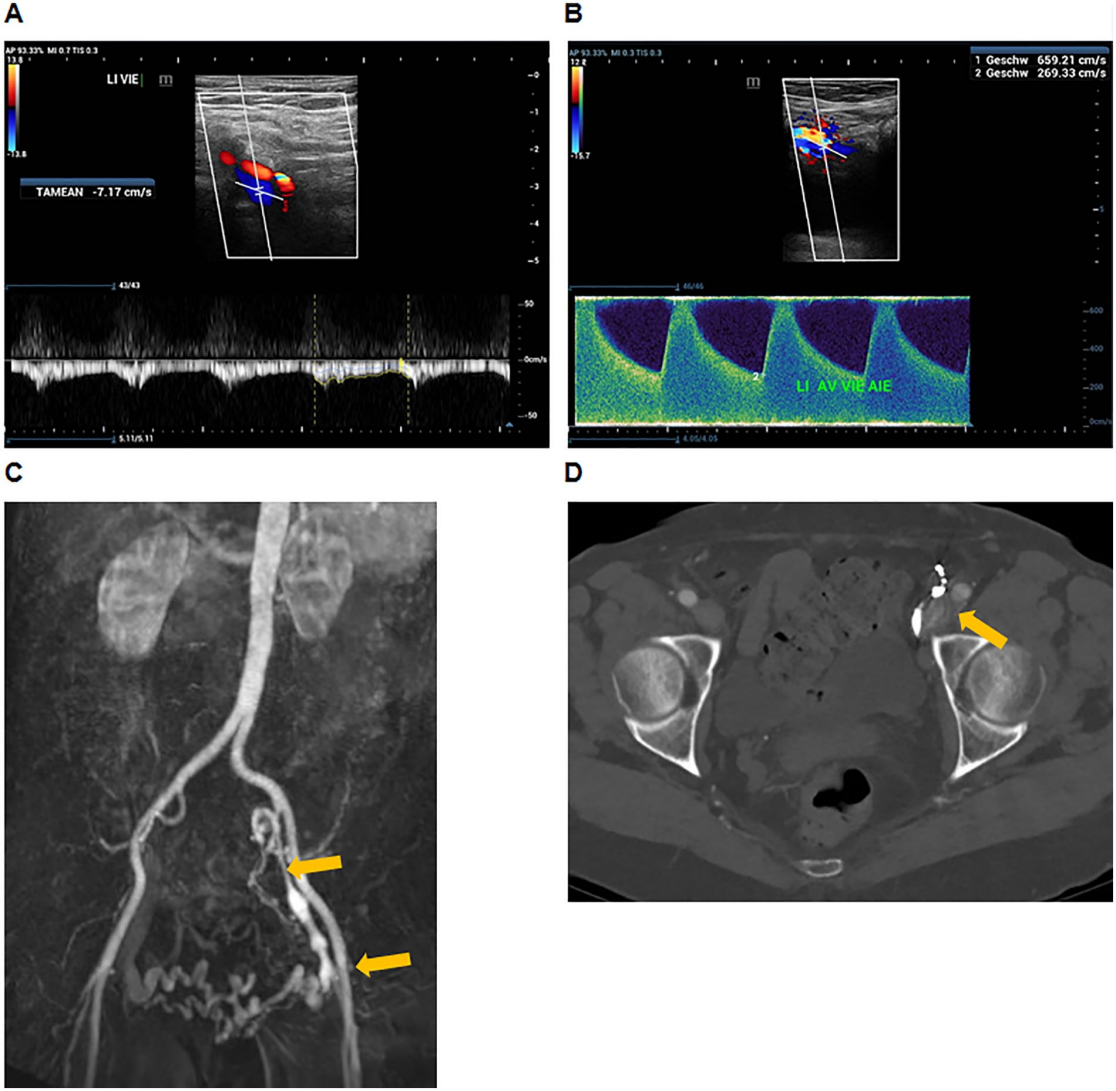
Recurrence of arteriovenous fistula (AVF) between the external iliac artery and venous collateral branches 1 month after coil embolization. (A) Pulsed wave doppler of the distal external iliac vein with arterial pulsatile flow modulation. (B) AVF flow profile from a connection between the external iliac artery and vein. (C) Magnetic resonance venography showing recurrence of AVF between the external and internal iliac artery and branches of the chronically diseased iliac veins (orange arrows). (D) Computed tomography angiography showing AVF between the external iliac artery and respective veins (orange arrow).

One month after this intervention, the patients returned for a clinical follow-up and imaging studies. Clinically, no significant improvement in leg swelling was observed. Imaging follow-up by duplex ultrasound, magnetic resonance venography, and computed tomography venography of the iliac veins again revealed the presence of multiple AVF channels between the arterial iliac axis and the post-thrombotically diseased iliac veins (Figure 3A–D).

## Second Approach

Due to the lack of sustainability of embolization of iliac AVF in the presence of a chronic iliac vein obstruction and according to the persisting clinical manifestation of venous hypertension, the patient was finally scheduled for direct iliac vein recanalization and stent implantation. Before the recanalization procedure, sufficient inflow from the ipsilateral FV and DFV was confirmed by pre-interventional duplex sonography.

## Second Procedure

**Figure 4.**
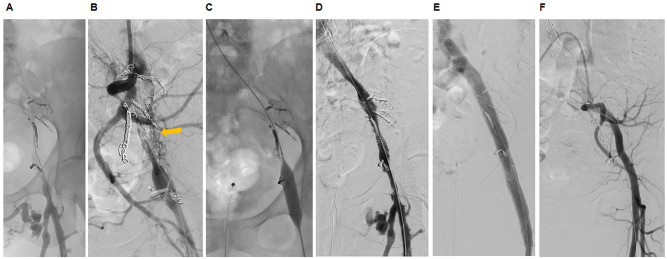
Second endovascular treatment approach with stent recanalization of the chronically occluded iliofemoral veins. (A) Venogram of the chronically occluded iliac veins (sheath in the ipsilateral femoral vein). (B) Selective angiography of left internal iliac artery showing recurrence of arteriovenous fistula (AVF) channels (orange arrow; cross-over sheath from the contralateral common femoral artery). (C) Pre-dilation with non-compliant high-pressure balloon of the chronically occluded iliac veins. (D) Venogram after pre-dilation showing continuous venous drainage via the iliac venous axis. (E) Final venogram after stent placement in the left common femoral vein, external iliac vein and common iliac vein. (F) Final selective angiogram of the left internal iliac artery showing successful closure of all AVF.

For stent recanalization of the chronically obstructed iliac veins, vascular access was gained via an ultrasound guided puncture of the left FV approximately 10 cm distal of the femoral vein confluence. Catheter-based venography demonstrated venous collaterals deriving from the scarred CFV and EIV (Figure 4A). To optimize fluoroscopic imaging of the iliocaval confluence, a second introducer sheath was inserted into the right CFV. Additional arterial access was used for intraprocedural imaging of the left sided iliac AVF. Therefore, a 45 cm cross-over sheath was inserted via the right sided common femoral artery and advanced into the left internal iliac artery (Figure 4B).

The iliac vein lesion was successfully crossed by using a 0.035″ stiff angled hydrophilic glidewire (Terumo Medical Corporation) and a 4 French angled hydrophilic support catheter (CXI, Cook Medical LLC). After confirmation of a correct in-line wire position by using lateral projections, the lesion was predilated (from distal/peripheral to proximal/central) with 12/40 mm, 14/40 mm, and 16/40 mm high-pressure non-compliant balloons (Atlas Gold, Bard BD) (Figure 4C and D). After determination of the stent landing zones by intravascular ultrasound, 3 venous stents were implanted into the left CFV, EIV, and CIV (from distal/peripheral to proximal/central: 12/80 mm [®Venovo, Bard BD], 14/150 mm [®BeYond, Bentley InnoMed GmbH], and 16/100 mm [®sinus-Obliquus, optimed Medizinische Instrumente GmbH]) (Figure 4E). The subsequent venogram, intravascular ultrasound, and external duplex ultrasound conjointly showed a satisfying morphological and hemodynamic result of the recanalized iliofemoral veins. Finally, the arterial angiogram confirmed the closure of iliac AVF (Figure 4F). At discharge, the patient was pain free, and there was a prompt resolution of the leg swelling. Regarding antithrombotic treatment, the patient was put on a therapeutic dose of low-molecular weight heparin for 2 weeks, which was then switched to apixaban 5 mg bid.

## Follow-up

**Figure 5.**
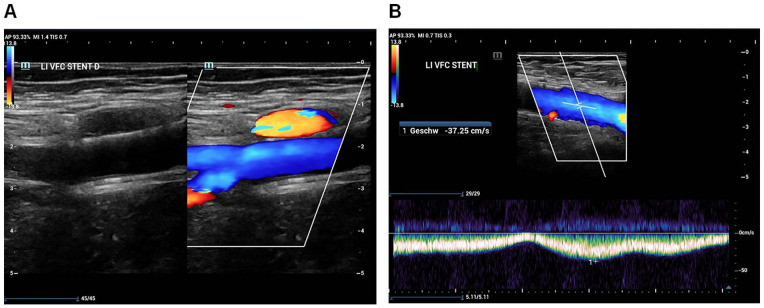
Duplex follow-up examination 6 months after iliofemoral venous recanalization and stent placement. (A) Duplex of the left femoral venous confluence (of the femoral and deep femoral vein) and the distal part of the stent in the common femoral vein. (B) Pulsed wave doppler of the left common femoral vein with regular venous flow modulation.

The patient came back for regular duplex ultrasound and clinical follow-up examinations after 1 week, 3 months, and 6 months. During follow-up, the patient reported a sustained reduction of symptoms (at 6 months Villalta scale 2 points) and duplex ultrasound confirmed patent venous stents as well as regular venous flow modulation (Figure 5).

## Discussion

The presented case demonstrates the potential association between post-thrombotic venous obstructions and the development of AVF as well as the efficacy of iliac venous stenting in the treatment of post-thrombotic AVF.

The detection of iliac AVF requires careful consideration of different potential etiologies^1^: apart from iatrogenic AVF following catheterization procedures or pelvic surgery, congenital iliac AVF warrants mention.^1,2^ Large congenital AVF potentially provoke massive cardiac dilation and ectasia of the inferior vena cava, which might be accompanied by high-output heart failure.^2^ In such cases, monitoring of cardiac imaging and function is essential. In the presented case, no clinical signs and symptoms of heart failure were observed, and the diameter of the inferior vena cava was within the normal range. In this patient, the temporal association with an iliofemoral deep vein thrombosis and the subsequent development of iliac AVF in the respective venous segment is highly indicative for the post-thrombotic development of the AVF.

Previous observations reported the rare occurrence of AVF in patients with a history of deep vein thrombosis.^3–6^ Referring to the presence of the post-thrombotic syndrome in the presented patient case, the history of deep vein thrombosis appears to be the most plausible origin for the occurrence of AVF. Other causes, such as previous endovascular or surgical procedures involving the respective region, could have been excluded. Furthermore, there was no history of puncture, trauma, or radiation involving the affected vascular segments.

The etiology of the development of AVF in patients with deep vein thrombosis is not fully clarified. Previous observations suggest an association between thrombosis, inflammation, and neovascularization, which conjointly result in venous remodeling and the formation of AVF.^4,7,8^ According to these observations, advanced patients’ age appears to be a risk factor for the development of post-thrombotic AVF. Whether an early thrombus removal strategy in the acute stage of iliofemoral deep vein thrombosis would have prevented the occurrence of post-thrombotic AVF in this specific case can only be speculated. Nevertheless, it should be noted that endovascular treatment of acute iliofemoral deep vein thrombosis potentially reduces severe manifestations of the post-thrombotic syndrome and should therefore be considered, taking into account the individual risk of bleeding.^9^

In the presence of chronic iliac vein occlusions, the development of post-thrombotic AVF substantially worsens the clinical signs and symptoms of venous hypertension, which is in line with our observation in the present case.^6^ Up to now, data on potential treatment options of post-thrombotic AVF are inconsistent.^3,5,6^ While the endovascular embolization of AVF has been reported to improve the clinical manifestation of venous hypertension, 1 small study reported high recurrence rates following embolization of post-thrombotic AVF.^3,5,6^ In the presented case, the visualization of iliac AVF by duplex ultrasound and magnetic resonance venography led to the primary decision for an embolization attempt. Since anticoagulation treatment was suspended after AVF embolization, we hypothesize that the failure of this treatment approach might be attributed to the development of new AVF rather than a reperfusion of previously embolized AVF. As suggested by previous reports, direct stent recanalization of the iliac veins resulted in an immediate closure of post-thrombotic AVF and corresponding clinical improvement.

The present observation, which demonstrated the successful and durable closure of post-thrombotic AVF by direct iliac vein recanalization and stent placement, supports previous reports on the superior durability of iliac venous stenting versus embolization in patients with post-thrombotic AVF.

## Conclusion

AVF are a rare manifestation of the post-thrombotic syndrome and potentially deteriorate clinical manifestation of venous hypertension. In patients with post-thrombotic iliac vein occlusion, direct endovascular recanalization and stent placement results in durable closure of AVF and clinical improvement.
